# Symptom profiles and their risk factors in patients with post-COVID-19 condition: a Dutch longitudinal cohort study

**DOI:** 10.1093/eurpub/ckad152

**Published:** 2023-08-22

**Authors:** Sander K R van Zon, Aranka V Ballering, Sandra Brouwer, Judith G M Rosmalen, for the Lifelines Corona Research Initiative, H Marike Boezen, Jochen O Mierau, H Lude Franke, Jackie Dekens, Patrick Deelen, Pauline Lanting, Judith M Vonk, Ilja Nolte, Anil P S Ori, Annique Claringbould, Floranne Boulogne, Marjolein X L Dijkema, Henry H Wiersma, Robert Warmerdam, Soesma A Jankipersadsing, Irene van Blokland, Geertruida H de Bock, Judith G M Rosmalen, Cisca Wijmenga

**Affiliations:** Department of Health Sciences, Community and Occupational Medicine, University of Groningen, University Medical Center Groningen, Groningen, The Netherlands; Department of Psychiatry, University of Groningen, University Medical Center Groningen, Groningen, The Netherlands; Department of Health Sciences, Community and Occupational Medicine, University of Groningen, University Medical Center Groningen, Groningen, The Netherlands; Department of Psychiatry, University of Groningen, University Medical Center Groningen, Groningen, The Netherlands; Department of Internal Medicine, University of Groningen, University Medical Center Groningen, Groningen, The Netherlands

## Abstract

**Background:**

To improve research and care for patients with post**-**COVID-19 condition more insight into different subtypes of post**-**COVID-19 condition and their risk factors is urgently needed. We aimed to identify risk factors of post**-**COVID-19 condition in general and for specific symptom profiles.

**Methods:**

This study is based on data collected within the Lifelines Coronavirus disease 2019 (COVID-19) cohort (*N* = 76 503). Mean pre- and post-SARS-CoV-2 infection symptom scores were compared to classify post**-**COVID-19 condition. Latent Profile Analysis was used to identify symptom profiles. Logistic and multinomial regression analyses were used to examine the association between demographic, lifestyle and health-related risk factors and post**-**COVID-19 condition, and symptom profiles, respectively.

**Results:**

Of the 3465 participants having had COVID-19, 18.5% (*n* = 642) classified for post**-**COVID-19 condition. Four symptom profiles were identified: muscle pain, fatigue, cardiorespiratory and ageusia/anosmia. Female sex was a risk factor for the muscle pain and fatigue profiles. Being overweight or obese increased risk for all profiles, except the fatigue profile. Having a chronic disease increased the risk for all profiles except the ageusia/anosmia profile, with the cardiorespiratory profile being only significant in case of multimorbidity. Being unvaccinated increased risk of the ageusia/anosmia profile.

**Conclusions:**

Findings from this study suggest that Severe acute respiratory syndrome coronavirus 2 (SARS-CoV-2) may trigger different pathophysiological mechanisms that may result in different subtypes of post**-**COVID-19 condition. These subtypes have shared and unique risk factors. Further characterization of symptom profiles and quantification of the individual and societal impact of specific symptom profiles are pressing challenges for future research.

## Introduction

After the acute phase of COVID-19, a substantial proportion of individuals continues to experience symptoms of a physical, psychological and/or cognitive nature.^1^^,^^2^ The World Health Organization defined the ongoing experience of such symptoms 3–5 months from the onset of COVID-19 as post**-**COVID-19 condition.^3^ A recent study among the general Dutch population, using pre- and post-SARS-CoV-2 infection symptom measurements and a non-infected control group, estimated that COVID-19 causes persistent symptoms in 12.7% of individuals, including chest pain, painful muscles and difficulties breathing 3–5 months from the onset of COVID-19.^4^ To improve care for patients with post**-**COVID-19 condition, we urgently need more insight into subtypes of post**-**COVID-19 condition and their shared and unique risk factors.^5^ This may help understand heterogeneity in underlying mechanisms and facilitate the development of new treatments, which has great public health relevance given the medical and societal impact and costs.

Most previous studies have identified risk factors for post**-**COVID-19 condition with the assumption that post**-**COVID-19 condition is a homogeneous disorder. Two systematic reviews showed that female sex, increasing age, the presence of comorbidity, identifying as being part of a minority ethnicity, and acute disease severity increase the risk for post**-**COVID-19 condition.^6^^,^^7^ One of these reviews^6^ included hospitalized and non-hospitalized populations, while the other^7^ focused on hospitalized patients only. Yet, in practice, the majority of people with COVID-19 is not hospitalized.^8^ Furthermore, in both systematic reviews, the risk of selection and attrition bias was high for the majority of included studies. Since the publication of these reviews, the aforementioned risk factors were confirmed among large general population studies from the UK.^9^^,^^10^ In addition, these studies showed that smoking, being overweight and obese and specific comorbidities were associated with post**-**COVID-19 condition. However, research using pre- and post-SARS-CoV-2 infection symptom measurements is necessary to validate risk factors of post**-**COVID-19 condition by excluding symptoms that are not attributable to COVID-19.

Furthermore, it seems likely that post**-**COVID-19 condition encompasses multiple subtypes, that have shared and unique risk factors and mechanisms. The idea of post**-**COVID-19 condition subtypes is supported by studies on symptom profiles.^9–13^ Findings from these studies vary considerably as these have identified between 2 symptom profiles^10^ and 13 symptom^11^ profiles. Importantly, these studies could not establish whether symptoms were new or severely increased after the acute phase of COVID-19 or were a mere continuation of pre-existing symptoms. This may be problematic as misclassification of post**-**COVID-19 condition may not only obscure observed symptom profiles, but also the associations between risk factors and symptom profiles.^14^

We aim to study previously identified risk factors for post**-**COVID-19 condition and to examine whether such risk factors are associated with specific post**-**COVID-19 condition symptom profiles.

## Methods

### Study design and sample

This study used data from the Lifelines COVID-19 cohort (*n* = 76 503; mean age 53.6 (SD = 13.0) years; 60.8% female).^15^ This cohort examines the physical, psychological and societal impacts of the COVID-19 pandemic and is a digital add-on study to Lifelines.^16^ Lifelines is a multi-disciplinary prospective population-based cohort study examining the health and health-related behaviours of 167 729 persons living in the North of the Netherlands. It employs investigative procedures in assessing the biomedical, socio-demographic, behavioural, physical and psychological factors which contribute to the health and disease of the general population. Extensive information on the cohort, its design considerations and recruitment procedures are described elsewhere.^15–17^ In this study, we included data of 27 consecutive measurements from March 2020 until July 2022. Data were collected weekly in March, April and May 2020, biweekly from June until August 2020, monthly from August 2020 until June 2021 and every 1–3 months from July 2021. The response rates per wave varied between 28% and 49%. Lifelines and its add-on studies were conducted according to the guidelines of the Declaration of Helsinki, and all procedures were approved by the Medical Ethics Committee of the University Medical Center Groningen (2007/152). All participants provided written informed consent.

In this study, we included participants with a COVID-19 diagnosis between March 2020 and April 2022 (*n* = 12 150). This allowed us to select participants with post**-**COVID-19 condition 90–150 days after SARS-CoV-2 infection.^3^ We only included participants for whom we could establish whether or not they had developed post**-**COVID-19 condition. We further excluded participants who did not join the Lifelines COVID-19 cohort during the first or second wave as information on several potential risk factors was only collected during these waves. Finally, we excluded participants with missing data on potential risk factors. Supplementary figure S1 gives a detailed overview of the sample selection. Characteristics of excluded (*n* = 8685) and included (*n* = 3465) participants were similar (Supplementary table S1).

### Symptoms

Assessed symptoms included headache, dizziness, chest pain, lower back pain, nausea, painful muscles, difficulties breathing, feeling hot/cold alternately, tingling extremities, feeling a lump in the throat, general tiredness, heavy arms/legs, pain when breathing, runny nose, sore throat, dry cough, wet cough, fever, diarrhoea, stomach pain, ageusia/anosmia, sneezing and itchy eyes. The first 12 symptoms are derived from the Symptom CheckList-90 Somatization (SCL-90 SOM) subscale, which has been recommended for large-scale studies^18^ and has been shown to have sufficient measurement invariance, making it suitable to assess symptom experience repeatedly over time.^19^ The remainder of symptoms was added since these were considered to be COVID-19 related at the start of the Lifelines COVID-19 cohort. Experienced symptoms were assessed using an ordinal five-point Likert scale (1 ‘not at all’ and 5 ‘extremely’) in the past 7/14 days. The time frame changed when the follow-up time between measurements increased.

### COVID-19

COVID-19 diagnosis was measured between March 2020 and April 2022 and in this study we only included participants’ first COVID-19 diagnosis. Between March 2020 and August 2020, a COVID-19 diagnosis was based on either a positive PCR test, antigen test or a physician’s diagnosis of COVID-19. The latter was included as the PCR testing regime in the Netherlands was strongly restricted until August 2020. From August 2020 onwards, we defined a COVID-19 diagnosis based on a positive PCR test at the Public Health Service, positive antigen tests at work or school, ‘testing for entry’ or another official institute. In line with a previous study in the Lifelines COVID-19 Cohort, self-tests were not included.^4^

### Post-COVID-19 condition

Post-COVID-19 condition was classified by comparing mean post**-**SARS-CoV-2 infection symptom scores 90–150 days after infection with mean pre-infection symptom scores at least 7 days before infection.^3^^,^^4^ When the mean post-infection symptom score of at least one symptom was higher than three (i.e. moderate severity) and at least one point increased compared to the mean pre-infection score, participants were classified as having post**-**COVID-19 condition.^4^ We based this classification on the 10 symptoms that we previously identified as core symptoms using data from the Lifelines COVID-19 cohort.^4^ These core symptoms were chest pain, painful muscles, difficulties breathing, feeling hot/cold alternately, tingling extremities, feeling a lump in the throat, general tiredness, heavy arms/legs, pain when breathing and ageusia/anosmia.

### Risk factors

Age was measured at the wave of SARS-CoV-2 infection and categorized into three age groups representing early adult life, mid-life and later life (i.e. 18–39, 40–59 and ≥60 years). Sex was categorized into male and female. Educational level was categorized into low, medium and high. Smoking was assessed with the question ‘Have you smoked in the last 7/14 days?’. Those answering ‘yes’ at one of the time points were categorized as smokers. BMI (in kg/m^2^) was calculated based on height previously collected in Lifelines, and self-reported weight from the questionnaire closest to SARS-CoV-2 infection and categorized into normal weight (BMI <25), overweight (BMI ≥25 to <30) and obesity (BMI ≥30). Chronic diseases were assessed in the first and second wave with single-item questions assessing the presence of 17 diseases. These were combined into cardiovascular disease, lung disease, diabetes, chronic muscle disease, autoimmune disease, psychiatric disorder and other disease (Supplement table S2). We also categorized participants as having no chronic disease, one chronic disease or multimorbidity. Vaccination status was measured from wave 18 onwards (start 25 February 2021) and categorized as fully vaccinated, partially vaccinated and unvaccinated at time of the SARS-CoV-2 infection. Participants with a SARS-CoV-2 infection until wave 18 were categorized as unvaccinated as the national vaccination campaign had not yet started.^20^ Hospitalization was assessed by the question ‘Have you been hospitalized for a COVID-19 infection?’ and was dichotomized into no/yes. We categorized participants as being infected by the Alpha (March 2020—July 2020), Delta (July 2020—January 2021) or Omicron (January 2021 to April 2022) variant based on the timing of their SARS-CoV-2 infection.^21^ Finally, we categorized the season of infection to correct for potential seasonal influences on symptom levels.

### Statistical analyses

Characteristics of the study population were described for the total study sample and separately for participants without and with post**-**COVID-19 condition. Latent Profile Analysis (LPA) using the ‘tidyLPA’ package in R Studio 2022.02.0 was used to identify symptom profiles within participants categorized as having post**-**COVID-19 condition.^22^ LPA is a data-driven technique that identifies profiles of individuals based on scoring patterns across continuous input variables.^23^ Four possible model configurations pertaining variance and covariance within and between classes were examined to see which set of parameters fit the data best. One to 10 classes were analysed to determine the optimal number of groups. Decisions regarding model fit and class solution were based on the Akaike information criterion (AIC), Bayesian information criterion (BIC), entropy (≥0.8), minimal probability of belonging to a class (≥0.9), minimal group size (≥5% of the analytic sample) the bootstrapped likelihood test (BLRT) *P* values and conceptual meaningfulness.^23^ For the AIC and BIC, lower values represent better model fit. Characterization of profiles was based on a mean symptom score of ≥2.5. Univariable and multivariable logistic regression analyses were performed to examine associations between potential risk factors and post**-**COVID-19 condition. For these analyses, specific chronic diseases were used. To examine whether participant characteristics were associated with the identified profiles, we performed a multivariable multinomial logistic regression analysis using the potential risk factors as independent variables and profile membership as dependent variable. Participants without persistent symptoms were the reference category. For this analysis, we used the count variable regarding chronic diseases. We did not include hospitalization as risk factor due to the low number of hospitalizations.

In sensitivity analyses, we categorized participants as having post**-**COVID-19 condition based on the 23 measured symptoms and repeated all analyses to enable comparing our results to studies using a broader definition of post**-**COVID-19 condition. As more participants classify for post**-**COVID-19 condition with a broader definition, we used the specific chronic diseases instead of the count variable in the multinomial logistic regression analysis. Second, we re-ran the multinomial regression analysis with the least severe profile (i.e. least symptoms ≥2.5) as the reference category instead of participants without post**-**COVID-19 condition.

All analyses, except LPA, were performed in IBM SPSS Statistics version 28.

## Results

### Study population

A total of 80 338 questionnaires were completed by 3465 participants (mean age 55.8 years, SD 11.6, 64.3% female). Participants completed a median of 25 questionnaires (IQR: 21-27). Of the 3465 participants, 18.5% (*n* = 642) classified for post**-**COVID-19 condition (Table 1; Supplementary table S3). Hereof, 59.5% (*n* = 382) was aged 40–59 years and 72.6% (*n* = 466) was female.

**Table 1:** Participant characteristics of the analytic study sample (*n* = 3465)

	Total study sample		COVID-19		Post-COVID-19 condition	
	(*n* = 3465)		(*n* = 2823)		(*n* = 642)	
Characteristic	*n*	%	*n*	%	*N*	%
Age						
18–39	343	9.9	282	10.0	61	9.5
40–59	1810	52.2	1428	50.6	382	59.5
≥60	1312	37.9	1113	39.4	199	31.0
Sex						
Male	1238	35.7	1062	37.6	176	27.4
Female	2227	64.3	1761	62.4	466	72.6
Educational level						
High	1185	34.2	964	34.1	221	34.4
Medium	1380	39.8	1102	39.0	278	43.3
Low	811	23.4	684	24.2	128	19.9
Unknown	89	2.6	74	2.6	15	2.3
Smoking						
No	3132	90.4	2551	90.4	581	90.5
Yes	333	9.6	272	9.6	61	9.5
Body mass index						
Healthy	1535	44.3	1304	46.2	231	36.0
Overweight	1386	40.0	1113	39.4	273	42.5
Obese	544	15.7	406	14.4	138	21.5
Chronic diseases						
Cardiovascular disease	345	10.0	258	9.1	87	13.6
Lung disease	336	9.7	248	8.8	88	13.7
Diabetes	88	2.5	60	2.1	28	4.4
Chronic muscle disease	46	1.3	26	0.9	20	3.1
Autoimmune disease	145	4.2	110	3.9	35	5.5
Psychiatric disorder	76	2.2	49	1.7	27	4.2
Other chronic disease	523	15.1	385	13.6	138	21.5
Chronic disease						
No chronic disease	2469	71.3	2080	73.7	389	60.6
One chronic disease	601	17.3	454	16.1	147	22.9
Multimorbidity	395	11.4	289	10.2	106	16.5
Vaccination status						
Fully vaccinated	1584	45.7	1330	47.1	254	39.6
Partially vaccinated	98	2.8	80	2.8	18	2.8
Not vaccinated	1783	51.5	1413	50.1	370	57.6
Virus variant						
Omicron	2656	76.7	2180	77.2	476	74.1
Delta	649	18.7	508	18.0	141	22.0
Alpha	160	4.6	135	4.8	25	3.9
Hospitalization						
No	3340	96.4	2737	97.0	603	93.9
Yes	125	3.6	86	3.0	39	6.1
Season of infection						
Winter	2555	73.7	2102	74.5	453	70.6
Spring	388	11.2	313	11.1	75	11.7
Summer	77	2.2	64	2.3	13	2.0
Autumn	445	12.8	344	12.2	101	15.7

### Symptom profiles

**Figure 1:**
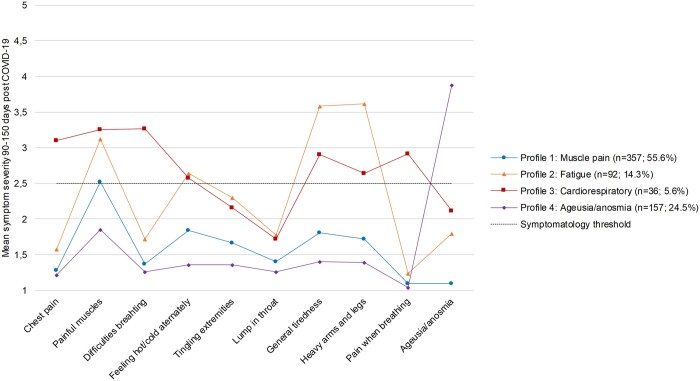
Mean symptom severity 90–150 days post**-**COVID-19 by symptom profile

A four-profile solution with model one was identified as most optimal based on model fit statistics and conceptual meaningfulness (Supplementary table S4). The muscle pain profile (*n* = 357; 55.6%) is characterized by a high mean symptom level of muscle pain (figure 1; Supplementary table S5). The fatigue profile (*n* = 92; 14.3%) is characterized by heavy arms/legs, general tiredness, painful muscles and feeling hot/cold alternately. The cardiorespiratory profile (*n* = 36; 5.6%) is characterized by difficulties breathing, painful muscles, chest pain, pain when breathing, general tiredness, heavy arms/legs and feeling hot/cold alternately. The ageusia/anosmia profile (*n* = 157; 24.5%) is characterized by ageusia/anosmia. Supplementary table S6 shows participant characteristics stratified by symptom profile.

### Risk factors

Multivariable logistic regression analysis showed that female sex, overweight and obesity, diabetes, chronic muscle disease, the category other chronic diseases, being unvaccinated and hospitalization for acute COVID-19 increased the risk for post**-**COVID-19 condition (table 2). A low educational level decreased the risk, just as participants most likely infected with the Alpha variant compared to Omicron. Age and smoking did not affect the risk for post**-**COVID-19 condition.

**Table 2:** Associations between participant characteristics and post**-**COVID-19 condition

			Univariable	Multivariable

	*n*	%	OR (95% CI)	OR (95% CI)
Age				
18–39	343	9.9	Ref	Ref
40–59	1810	52.2	1.24 (0.92, 1.67)	1.12 (0.82, 1.53)
≥60	1312	37.9	0.83 (0.60, 1.13)	0.78 (0.56, 1.10)
Sex				
Male	1238	35.7	Ref	Ref
Female	2227	64.3	1.60 (1.32, 1.93)	1.56 (1.28, 1.90)
Educational level				
High	1185	34.2	Ref	Ref
Medium	1380	39.8	1.10 (0.90, 1.34)	0.95 (0.77, 1.16)
Low	811	23.4	0.82 (0.64, 1.04)	0.71 (0.55, 0.91)
Unknown	89	2.6	0.88 (0.50, 1.57)	0.81 (0.44, 1.46)
Smoking				
No	3132	90.4	Ref	Ref
Yes	333	9.6	0.98 (0.73, 1.32)	0.96 (0.71, 1.30)
Body mass index				
Healthy	1535	44.3	Ref	Ref
Overweight	1386	40.0	1.38 (1.14, 1.68)	1.42 (1.17, 1.74)
Obese	544	15.7	1.92 (1.51, 2.44)	1.66 (1.29, 2.14)
Cardiovascular disease				
No	3120	90.0	Ref	Ref
Yes	345	10.0	1.56 (1.20, 2.02)	1.26 (0.95, 1.69)
Lung disease				
No	3129	90.3	Ref	Ref
Yes	336	9.7	1.65 (1.27, 2.14)	1.28 (0.96, 1.69)
Diabetes				
No	3377	97.5	Ref	Ref
Yes	88	2.5	2.10 (1.33, 3.32)	1.74 (1.06, 2.84)
Chronic muscle disease				
No	3419	98.7	Ref	Ref
Yes	46	1.3	3.46 (1.92, 6.24)	2.19 (1.16, 4.13)
Autoimmune disease				
No	3320	95.8	Ref	Ref
Yes	145	4.2	1.42 (0.96, 2.10)	0.99 (0.65, 1.51)
Psychiatric disorder				
No	3389	97.8	Ref	Ref
Yes	76	2.2	2.48 (1.54, 4.01)	1.66 (0.99, 2.75)
Other chronic disease				
No	2942	84.9	Ref	Ref
Yes	523	15.1	1.73 (1.40, 2.15)	1.42 (1.12, 1.80)
Vaccination status				
Fully vaccinated	1584	45.7	Ref	Ref
Partially vaccinated	98	2.8	1.18 (0.69, 2.00)	1.11 (0.64, 1.94)
Not vaccinated	1783	51.5	1.37 (1.15, 1.64)	1.29 (1.03, 1.62)
Virus variant				
Omicron	2656	76.7	Ref	Ref
Delta	649	18.7	1.27 (1.03, 1.57)	0.95 (0.68, 1.33)
Alpha	160	4.6	0.85 (0.55, 1.31)	0.46 (0.26, 0.81)
Hospitalization				
No	3340	96.4	Ref	Ref
Yes	125	3.6	2.06 (1.40, 3.04)	2.34 (1.49, 3.65)
Season of infection				
Winter	2555	73.7	Ref	Ref
Spring	388	11.2	1.11 (0.85, 1.46)	1.01 (0.71, 1.45)
Summer	77	2.2	0.94 (0.51, 1.73)	0.67 (0.34, 1.30)
Autumn	445	12.8	1.36 (1.07, 1.74)	1.21 (0.87, 1.70)

Female sex was a risk factor for the muscle pain and fatigue profiles while age was not a risk factor for any of the profiles (table 3). A low educational level was protective for the cardiorespiratory and ageusia/anosmia profiles. For lifestyle-related factors, being overweight or obese increased the risk for all profiles except the fatigue profile, while smoking was not a risk factor for any of the profiles. Regarding health status, having a chronic disease increased the risk for all profiles except the ageusia/anosmia profile, with the cardiorespiratory profile being only significant in case of multimorbidity. Finally, being unvaccinated increased the risk for the ageusia/anosmia profile, whereas the Alpha variant was associated with a decreased risk in this profile compared to Omicron.

**Table 3:** Multivariable multinomial logistic regression analysis of determinants for profile status with participants without post**-**COVID-19 condition (*n* = 2749) as the reference group^a^

	Muscle pain (*n* = 349)	Fatigue (*n* = 92)	Cardiorespiratory (*n* = 35)	Ageusia/anosmia (*n* = 151)

	OR (95% CI)	OR (95% CI)	OR (95% CI)	OR (95% CI)
Age				
18–39	Ref	Ref	Ref	Ref
40–59	1.03 (0.68, 1.55)	0.98 (0.47, 2.04)	3.94 (0.52, 29.7)	1.44 (0.75, 2.76)
≥60	0.77 (0.48, 1.16)	0.72 (0.33, 1.61)	1.46 (0.17, 12.4)	1.10 (0.55, 2.20)
Sex				
Male	Ref	Ref	Ref	Ref
Female	1.63 (1.26, 2.10)	2.09 (1.24, 3.51)	1.67 (0.76, 3.64)	1.10 (0.78, 1.57)
Educational level				
High	Ref	Ref	Ref	Ref
Medium	1.11 (0.85, 1.44)	0.99 (0.61, 1.62)	0.46 (0.22, 0.98)	0.78 (0.54, 1.13)
Low	0.85 (0.62, 1.18)	0.95 (0.53, 1.72)	0.30 (0.10, 0.84)	0.53 (0.33, 0.87)
Smoking				
No	Ref	Ref	Ref	Ref
Yes	0.72 (0.47, 1.11)	1.53 (0.82, 2.82)	1.19 (0.40, 3.53)	1.07 (0.61, 1.88)
Body mass index				
Healthy	Ref	Ref	Ref	Ref
Overweight	1.53 (1.18, 1.98)	0.76 (0.46, 1.24)	3.08 (1.18, 8.08)	1.62 (1.12, 2.36)
Obese	1.77 (1.28, 2.44)	1.24 (0.71, 2.17)	6.28 (2.29, 17.2)	1.82 (1.12, 2.98)
Chronic disease				
No	Ref	Ref	Ref	Ref
One	1.78 (1.35, 2.35)	2.31 (1.39, 3.86)	2.16 (0.90, 5.22)	1.21 (0.78, 1.88)
Multimorbidity	1.82 (1.31, 2.54)	3.13 (1.80, 5.45)	4.77 (2.09, 10.9)	1.05 (0.60, 1.84)
Vaccination status				
Fully vaccinated	Ref	Ref	Ref	Ref
Not vaccinated	1.01 (0.76, 1.33)	1.38 (0.81, 2.35)	2.23 (0.95, 5.27)	2.45 (1.65, 3.79)
Virus variant				
Omicron	Ref	Ref	Ref	Ref
Delta	0.79 (0.51, 1.22)	1.41 (0.71, 2.78)	0.70 (0.21, 2.39)	1.00 (0.59, 1.69)
Alpha	0.87 (0.45, 1.67)	0.42 (0.12, 1.55)	2.81 (0.49, 16.2)	0.22 (0.05, 0.99)
Season				
Winter	Ref	Ref	Ref	Ref
Spring/summer	1.02 (0.68, 1.54)	1.44 (0.75, 2.75)	0.48 (0.11, 2.15)	0.81 (0.45, 1.47)
Autumn	1.19 (0.76, 1.87)	1.02 (0.48, 2.14)	1.58 (0.45, 5.61)	1.37 (0.79, 2.40)

a Due to a low number of participants in some cells: unknown education excluded; partly vaccinated combined with fully vaccinated; spring and summer combined.

### Sensitivity analyses

Using 23 symptoms, 1024 (29.5%) participants classified for post**-**COVID-19 condition (Supplementary tables S7 and S8). Profile solutions were similar, although the muscle pain profile became an unspecific symptom profile (Supplementary tables S9–S11). Logistic regression analyses showed similar results as well (Supplementary table S12). Multinomial regression analyses, most importantly, showed that chronic muscle disease was a risk factor for the fatigue and cardiorespiratory profiles, lung- and auto-immune diseases were risk factors for the cardiorespiratory profile and diabetes for the fatigue profile (Supplementary table S13). Hospitalization increased the risk for the fatigue and cardiorespiratory profiles. Multinomial regression analysis with the muscle pain profile as the reference category showed that smoking was positively associated with the fatigue profile, obesity and multimorbidity with the cardiorespiratory profile and not being vaccinated with the ageusia/anosmia profile (Supplementary table S14). Overweight and a lower educational level were negatively associated with the fatigue and cardiorespiratory profile, respectively.

## Discussion

This study identified four symptom profiles for post**-**COVID-19 condition with shared and unique risk factors, suggesting that SARS-CoV-2 may trigger different pathophysiological mechanisms that may lead to different subtypes of post**-**COVID-19 condition. Patients in the seemingly most severe profile, i.e. the cardiorespiratory profile, had the highest likelihood of being overweight, obese and having multimorbidity.

The following limitations should be acknowledged before interpreting the results of this study. First, consistent longitudinal data on cognitive symptoms and post-exertional malaise were not available while these symptoms are potentially relevant for post**-**COVID-19 condition.^24^ Second, there may be some selection bias as participants in the Lifelines COVID-19 cohort were older and more often female compared to the general Lifelines population. Third, misclassification of post**-**COVID-19 condition may have occurred as an increase in symptoms may be related to other causes.^4^ As reasons for symptom increases in those potentially misclassified are likely heterogeneous, we do not expect important differences in results if misclassification could completely be avoided. Fourth, the small sample size of the cardiorespiratory profile and within some of the variable categories may lead to considerable uncertainty around some estimates and potentially false non-significant findings. Fifth, the virus variant was assigned based on the dominant variant at wave of infection.^21^ Some misclassification is therefore not ruled out. Sixth, while we adjusted analyses for vaccination status we did not take type and timing of vaccination into account, while this may be important for the development of post**-**COVID-19 condition.^25^^,^^26^

A major strength of this study is the use of longitudinal data from 27 consecutive waves since the start of the pandemic. This allows for the calculation of pre-COVID-19 symptom severity, enabling us to classify participants as developing post**-**COVID-19 condition based on increased or new symptoms, reducing the risk of misclassification.^14^ In addition, the study sample completed a median of 25 questionnaires, which is relatively high. Finally, the SCL-90 SOM subscale is a validated instrument, suitable for assessing symptoms in large-scale cohort studies over time.

In line with previous studies, we found that female sex,^6^^,^^7^^,^^9^^,^^10^ weight status,^9^^,^^10^ comorbidity,^6^^,^^7^^,^^9^ being unvaccinated^27^ and hospitalization^10^ were determinants of post**-**COVID-19 condition. In contrast to previous studies, older age,^9^^,^^10^ smoking,^9^^,^^10^ cardiovascular disease, lung disease and psychiatric disorder^9^ were not significantly associated with post**-**COVID-19 condition, although point estimates for cardiovascular disease, lung disease and psychiatric disorder were increased. The relatively low percentage of COVID-19 cases may be explained by the low prevalence in the Northern Netherlands during the initial stage of the pandemic,^15^ the drop-out during the first phase of the pandemic and the fact that we did not include positive self-tests. While we most likely underestimate the prevalence of COVID-19, we expect that this did not influence the associations and profiles. While drop-out may have been selective in the entire Lifelines COVID-19 cohort, characteristics of those excluded (*n* = 8450) from our initial sample of 12 150 participants with COVID-19 were similar to the characteristics of those included in the analytic study sample.

We further found that participants most likely infected with the Alpha variant were at lower risk to develop post**-**COVID-19 condition compared to Omicron. Recently, a British study showed that people with Omicron had a lower risk to develop post**-**COVID-19 condition than people with the Delta variant.^25^ While we found that Omicron reduced the risk in a univariable analysis, this result was not maintained in our multivariable analysis. Differences in classification of post**-**COVID-19 condition, covariate adjustment and selection of participants may explain this difference. The proportion of fully vaccinated participants was lower in the analytic study sample than in those excluded. This can be explained by the increased time between measurement waves later in the pandemic in combination with the increase in vaccinations. Since this is thus directly related to the study design, we do not expect it to influence the validity of the findings.

Among 642 participants with post**-**COVID-19 condition, we identified four symptom profiles that may represent subtypes of post**-**COVID-19 condition. In line with previous studies,^9–13^ we found two symptom profiles that were characterized by general tiredness, heavy limbs and painful muscles. One of these profiles was characterized by the additional cardiorespiratory symptoms chest pain, difficulties breathing and pain while breathing. While this concurs with a meta-regression analysis,^13^ separate profiles for respiratory symptoms have been identified as well.^10^^,^^11^^,^^13^ Differences in sample size and study design compared to previous studies may explain why we did not find a separate respiratory profile. In contrast to previous studies, neither in the main analyses including previously defined core symptoms^4^ nor in sensitivity analyses did we identify profiles characterized by upper respiratory symptoms like cough or sore throat.^9^^,^^11^ This may indicate that previous studies could not distinguish between symptoms attributable to COVID-19, and those being a mere continuation of pre-existing symptoms. Finally, in line with studies from the UK^12^ and Germany^11^ we found a clear profile of patients experiencing solely ageusia/anosmia with few additional symptoms. Predictors for post**-**COVID-19 condition and for belonging to a more severe symptom profile mainly include excessive weight status and pre-existing health conditions. Excessive weight status and existing health conditions seem biological plausible risk factors for post**-**COVID-19 condition as they affect e.g. the immune, endocrine, circulatory and respiratory systems. Findings highlight the public health relevance of a healthy lifestyle and the prevention of overweight and chronic diseases.

In the current study, the cardiorespiratory profile seems to represent a small group of patients with a multitude of debilitating symptoms. The finding that the risk increase by overweight, obesity and multimorbidity is more pronounced for this particular profile than for the other profiles, indicates that existing pathology might contribute to a more severe subtype of post**-**COVID-19 condition. Similar results regarding weight status were observed in the UK, where overweight and obese COVID-19 patients were also at risk to develop persistent respiratory symptoms after COVID-19.^9^ Although the cardiorespiratory profile represents only 1% of our total study sample, the consequences on a population level could be substantial if indeed one in every hundred COVID-19 patients develops such a severe combination of persistent symptoms. Our findings regarding the size of symptom profiles are supported by a meta-regression analysis on three pre-defined symptom clusters, showing that 3.2% and 3.7% of individuals with symptomatic SARS-CoV-2 infection experienced persistent fatigue with bodily pain and ongoing respiratory problems, respectively.^13^

Our results suggest that it is important to distinguish between subtypes of post**-**COVID-19 condition when examining underlying mechanisms, as risk factors vary across subtypes. Future research should aim to disentangle how different profiles can be further characterized based on pathophysiological markers, preferably using pre- and post-SARS-CoV-2 measurements and a control group. It may be hypothesized that the cardiorespiratory profile is characterized by underlying organ damage in e.g. the lungs and heart,^1^ whereas the muscle pain and fatigue profiles may resemble known post-infectious somatic syndromes. Long term consequences regarding well-being and social participation may vary as well.^11^^,^^28^ One of the most pressing challenges is to further quantify the impact of specific symptom profiles on well-being and social participation, including work, in non-biased studies that are representative for the general population.

## Supplementary Material

ckad152_Supplementary_Data

## Data Availability

The data underlying this article were provided by Lifelines. Lifelines is a facility that is open for all researchers. Information on application and data access procedure is summarized on www.lifelines.nl.
