# Supplementary material for: Symptom profiles and their risk factors in patients with post-COVID-19 condition: a Dutch longitudinal cohort study
Source: Eur J Public Health. 2023 Aug 22;33(6):1163–70. doi: 10.1093/eurpub/ckad152 (PMC10710342; doi:10.1093/eurpub/ckad152)
Supplement: ckad152_Supplementary_Data [file ckad152_supplementary_data.pdf]

Supplement table 1. Comparison of characteristics between included and excluded individuals

			**Excluded by specific reason**												**Excluded total**	
	**Included participants N=3,465**		**Positive on first wave N=386**		**Positive on last wave N=756**		**No measurement 7 days before SARS-CoV-2 infection N=405**		**No measurement 90-150 days after SARS-CoV-2 infection* N=4,067**		**Incomplete symptom data N=110**		**Drop-out after wave of SARS-CoV-2 infection N= 2,726**		**Excluded participants N=8,450**	
Characteristic	n	%	n	%	n	%	n	%	n	%	n	%	n	%	n	%
Age																
18-39	343	9.9	89	23.1	69	9.1	84	20.7	404	9.9	10	9.1	389	14.3	1,045	12.4
40-59	1,810	52.2	213	55.2	404	53.4	248	61.2	2,103	51.7	62	56.4	1,521	55.8	4,551	53.9
≥60	1,312	37.9	84	21.8	283	37.4	73	18.0	1,560	38.4	38	34.5	816	29.9	2,854	33.8
Sex																
Male	1,238	35.7	118	30.6	273	36.1	138	34.1	1,502	36.9	33	30.0	909	33.3	2,973	35.2
Female	2,227	64.3	268	69.4	483	63.9	267	65.9	2,565	63.1	77	70.0	1,817	66.7	5,477	64.8
Educational level**																
High	1,185	34.2	148	38.3	293	38.8	126	31.1	1,390	34.2	35	31.8	1,027	37.7	3,019	35.7
Medium	1,380	39.8	145	37.6	306	40.5	162	40.0	1,605	39.5	47	42.7	1,112	40.8	3,377	40.0
Low	811	23.4	76	19.7	144	19.0	94	23.2	946	23.3	>25	>22.7	489	17.9	1,775	21.0
Unknown	89	2.6	17	4.4	13	1.7	23	5.7	126	3.1	<10	<9.1	98	3.6	279	3.3
Smoking																
No	3,132	90.4	339	87.8	689	91.1	291	88.4	3,686	90.7	97	88.2	2,422	89.5	7,524	90.1
Yes	333	9.6	47	12.2	67	8.9	38	11.6	380	9.3	13	11.8	285	10.5	830	9.9
Body Mass index																
Healthy	1,535	44.3	170	45.0	380	50.3	161	42.8	1,825	44.9	59	53.6	1,212	44.5	3,637	45.3
Overweight	1,386	40.0	135	35.7	261	34.5	135	35.9	1,602	39.4	37	33.6	1,062	39.0	3,097	38.6
Obese	544	15.7	73	19.3	115	15.2	80	21.3	638	15.7	14	12.7	451	16.6	1,298	16.2
Chronic disease																
No chronic disease	2,469	71.3	251	65.0	470	69.9	143	67.5	2,645	70.9	73	67.6	1,708	72.0	5,290	70.7
One chronic disease	601	17.3	68	17.6	131	19.5	37	17.5	661	17.7	15	13.9	396	16.7	1,308	17.5
Multimorbidity	395	11.4	67	17.4	71	10.6	32	15.1	425	11.4	20	18.5	267	11.3	882	11.8
Chronic diseases																

Cardiovascular disease	345	10.0	42	10.9	65	9.7	20	9.4	376	10.1	12	11.1	215	9.1	730	9.8
Lung disease	336	9.7	47	12.2	45	6.7	24	11.3	346	9.3	14	13.0	230	9.7	706	9.4
Diabetes	88	2.5	18	4.7	27	4.0	<10	<2.5	93	2.5	<10	<9.1	64	2.7	213	2.8
Chronic muscle disease	46	1.3	<10	<2.6	<10	<1.3	<10	<2.5	40	1.1	<10	<9.1	25	1.1	85	1.1
Autoimmune disease	145	4.2	26	6.7	31	4.6	14	6.6	181	4.9	11	10.2	121	5.1	384	5.1
Psychiatric disorder	76	2.2	14	3.6	10	1.5	11	5.2	85	2.3	<10	<9.1	52	2.2	176	2.4
Other chronic disease	523	15.1	85	22.0	111	16.5	42	19.8	574	15.4	17	15.7	343	14.5	1,172	15.7
Vaccination status**																
Fully vaccinated	1,584	45.7	0	0.0	611	80.8	0	0.0	2,712	66.7	19	17.3	2,094	76.8	5,436	64.3
Partially vaccinated	98	2.8	0	0.0	<10	<1.3	<10	<2.5	139	3.4	0	0.0	104	3.8	249	2.9
Not vaccinated	1,783	51.5	386	100	>135	>17.9	>400	>99.0	1,211	29.8	91	82.7	526	19.3	2,758	32.6
Virus variant**																
Omicron	2,656	76.7	0	0.0	756	100	28	6.9	3,738	91.9	>20	>18.2	2,612	95.8	7,159	84.7
Delta	649	18.7	0	0.0	0	0.0	72	17.8	241	5.9	<10	<9.1	97	3.6	416	4.9
Alpha	160	4.6	386	100	0	0.0	305	75.3	88	2.2	79	71.8	17	0.6	875	10.4
Hospitalization**																
No	3,340	96.4	363	94	756	100	373	92.1	3,955	97.2	>100	>90.9	2,675	98.1	8,223	97.3
Yes	125	3.6	23	6.0	0	0.0	32	7.9	112	2.8	<10	<9.1	51	1.9	227	2.7

* There was no possibility for individuals at wave 20 (n=353) and wave 26 (n=3,213) to be measured 90-150 days after SARS-CoV-2 infection, as there was no measurement wave that matched this time-window

** As some categories contained less than 10 participants, uncertainty was added to another category to prevent traceability of participants

Supplement table 2. Categorization of chronic diseases

	Answer categories to the question "Do you have a chronic disease?"	Final list of chronic diseases
1	Cardiovascular disease	Cardiovascular disease
2	High blood pressure	Cardiovascular disease
3	Heart attack	Cardiovascular disease
4	Narrowing of the arteries in the legs	Cardiovascular disease
5	Stroke or TIA	Cardiovascular disease
6	Other heart and/or coronary disease	Cardiovascular disease
7	Lung disease, such as asthma, COPD or chronic bronchitis	Lung disease
8	Liver disease*	Other
9	Kidney disease or reduced kidney function*	Other
10	Diabetes	Diabetes
11	Chronic muscle disease	Chronic muscle disease
12	Auto-immune disease, such as inflammatory bowel disorder or rheumatoid arthritis	Autoimmune disease
13	Cancer*	Other
14	Neurological disease, such as dementia or Parkinson's disease*	Other
15	Problems with your spleen*	Other
16	Psychiatric disorder	Psychiatric disorder
17	Other	Other

* Participants with "liver disease (n=11)", "kidney disease (n=25)", "neurological disease (n=11)", cancer (n=39) and "problems with spleen (n=5)" were merged with the "other" disease group as these numbers are too low for the multivariable regression analyses.

Supplement table 3. Mean (SD) symptom severity, presence of symptoms at moderate severity, and presence of symptoms with a substantial increase of symptom severity (i.e. 1-point increase) to at least moderate severity 90-150 days after infection in those with COVID-19 and post COVID-19 condition

	COVID-19 (n=2,823)					Post COVID-19 condition (n=624)				
	Mean (SD)	Presences of symptoms		Substantial increase of symptom severity		Mean (SD)	Presences of symptoms		Substantial increase of symptom severity	
		n	%	n	%		n	%	n	%
Chest pain	1.08 (0.28)	<10	<0.3	-	-	1.41 (0.71)	56	8.7	51	7.9
Painful muscles	1.43 (0.58)	92	3.3	-	-	2.48 (1.10)	301	46.9	242	37.7
Difficulties breathing	1.08 (0.28)	<10	<0.3	-	-	1.50 (0.81)	71	11.1	64	10.0
Feeling hot and cold alternately	1.17 (0.40)	20	0.7	-	-	1.88 (1.10)	160	24.9	134	20.9
Tingling extremities	1.16 (0.38)	23	0.8	-	-	1.71 (0.97)	121	18.8	100	15.6
Feeling a lump in the throat	1.10 (0.30)	<10	<0.3	-	-	1.44 (0.83)	64	10.0	59	9.2
General tiredness	1.18 (0.39)	13	0.5	-	-	2.03 (1.09)	180	28.0	164	25.5
Heavy arms and legs	1.15 (0.37)	12	0.4	-	-	1.96 (1.05)	168	26.2	143	22.3
Pain when breathing	1.02 (0.14)	0	0.0	-	-	1.20 (0.55)	22	3.4	22	3.4
Ageusia/anosmia	1.07 (0.29)	<10	<0.3	-	-	1.94 (1.37)	190	29.6	185	28.8

Supplement table 4. Fit statistics for the Latent Profile Analysis

Model	Classes	AIC	BIC	Entropy	Minimal probability	Maximal probability	N min	N max	BLRT-p
1	1	17321.21	17410.50	1.00	1.00	1.00	1.00	1.00	
1	2	16801.14	16939.54	0.98	0.99	0.99	0.29	0.71	0.01
1	3	16054.94	16242.45	0.95	0.97	0.98	0.09	0.64	0.01
1	4	15763.92	16000.54	0.90	0.87	0.98	0.06	0.56	0.01
1	5	15611.12	15896.85	0.92	0.89	0.99	0.03	0.51	0.01
1	6	14994.67	15329.51	0.94	0.85	1.00	0.02	0.49	0.01
1	7	14932.31	15316.26	0.91	0.80	1.00	0.03	0.42	0.01
1	8	14904.96	15338.03	0.91	0.77	1.00	0.02	0.41	0.01
1	9	14833.92	15316.09	0.92	0.77	1.00	0.02	0.39	0.01
1	10	14833.13	15364.42	0.88	0.70	1.00	0.03	0.32	0.13
2	1	16146.63	16436.83	1.00	1.00	1.00	1.00	1.00	
2	2	15677.50	16016.81	0.98	0.99	1.00	0.29	0.71	0.01
2	3	15642.34	16030.76	0.81	0.88	1.00	0.29	0.38	0.01
2	4	15286.27	15723.80	0.92	0.88	0.99	0.10	0.49	0.01
2	5	15035.55	15522.19	0.93	0.88	0.99	0.09	0.44	0.01
2	6	14953.75	15489.50	0.93	0.87	0.99	0.07	0.43	0.01
2	7	14909.46	15494.32	0.91	0.86	1.00	0.07	0.34	0.01
2	8	14866.78	15500.76	0.91	0.84	1.00	0.01	0.34	0.01
2	9	14323.88	15006.96	0.92	0.87	1.00	0.01	0.30	0.01
2	10	14283.96	15016.16	0.92	0.86	1.00	0.01	0.28	0.01
3	Could not be estimated								
4	Could not be estimated								

Model 1: variance within class, no variance between classes, no covariance within class, no covariance between classes
Model 2: variance within class, no variance between classes, covariance within class, no covariance between classes
Model 3: variance within class, variance between classes, no covariance within class, no covariance between classes
Model 4: variance within class, variance between classes, covariance within class, covariance between classes
Abbreviations: AIC: Akaike information criterion; BIC: Bayesian information criterion; BLRT: bootstrapped likelihood test

Supplement table 5. Mean (SD) symptom severity and presence of symptoms with a substantial increase of symptom severity (i.e. 1-point increase) to at least moderate severity 90-150 days after infection by symptom profile

	**No post COVID-19 (n=2,823)**	**Muscle pain (n=357)**			**Fatigue (n=92)**			**Cardiorespiratory (n=36)**			**Ageusia/anosmia (n=157)**		
	Mean (SD)	Mean (SD)	Substantial increase		Mean (SD)	Substantial increase		Mean (SD)	Substantial increase		Mean (SD)	Substantial increase	
			n	%		n	%		n	%		n	%
Chest pain	1.09 (0.28)	1.28 (0.54)	15	4.2	1.58 (0.70)	<10	<10.7	3.10 (0.90)	25	69.4	1.21 (0.41)	<10	<6.4
Painful muscles	1.43 (0.58)	2.52 (1.04)	163	45.7	3.12 (1.14)	41	44.6	3.25 (1.11)	19	52.8	1.85 (0.84)	19	12.1
Difficulties breathing	1.08 (0.28)	1.37 (0.66)	25	7.0	1.72 (0.85)	11	12.0	3.26 (0.89)	24	66.7	1.26 (0.49)	<10	<6.4
Feeling hot/cold alternately	1.17 (0.40)	1.84 (1.03)	76	21.3	2.64 (1.33)	38	41.3	2.57 (1.29)	14	38.9	1.36 (0.69)	<10	<6.4
Tingling extremities	1.16 (0.38)	1.66 (0.90)	59	16.5	2.30 (1.15)	25	27.2	2.16 (1.19)	10	27.8	1.36 (0.73)	<10	<6.4
Feeling a lump in the throat	1.10 (0.30)	1.40 (0.78)	29	8.1	1.77 (1.06)	19	20.7	1.72 (1.19)	<10	<27.8	1.26 (0.61)	<10	<6.4
General tiredness	1.18 (0.39)	1.81 (0.83)	66	18.5	3.58 (0.87)	74	80.4	2.90 (1.31)	15	41.7	1.40 (0.64)	<10	<6.4
Heavy arms and legs	1.15 (0.37)	1.72 (0.76)	52	14.6	3.62 (0.77)	74	80.4	2.64 (1.18)	11	30.6	1.39 (0.63)	<10	<6.4
Pain when breathing	1.02 (0.14)	1.09 (0.29)	<10	<2.8	1.24 (0.42)	0	0.0	2.91 (0.88)	21	58.3	1.04 (0.18)	0	0.0
Ageusia/anosmia	1.08 (0.29)	1.10 (0.34)	<10	<2.8	1.80 (1.17)	18	19.6	2.11 (1.54)	10	27.8	3.88 (0.82)	153	97.5

Supplement table 6. Participant characteristics per symptom profile

	**Muscle pain (n=357)**		**Fatigue (n=92)**		**Cardiorespiratory (n=36)**		**Ageusia/ Anosmia (n=157)**	
	N	%	N	%	N	%	n	%
Age								
18-39	37	10.4	<10	<10.9	<10	<27.8	13	8.3
40-59	205	57.4	54	58.7	27	75.0	96	61.1
≥60	115	32.2	29	31.5	<10	<27.8	48	30.6
Sex								
Male	92	25.8	19	20.7	<10	<27.8	56	35.7
Female	265	74.2	73	79.3	27	75.0	101	64.3
Educational level								
High	113	31.7	30	32.6	17	47.2	61	38.9
Medium	162	45.4	40	43.5	13	36.1	63	40.1
Low	74	20.7	22	23.9	<10	<27.8	27	17.2
Unknown	<10	<2.8	0	0.0	<10	<27.8	<10	<6.4
Smoking								
No	329	92.2	79	85.9	32	88.9	141	89.8
Yes	28	7.8	13	14.1	<10	<27.8	16	10.2
Body Mass index								
Healthy	125	35.0	43	46.7	<10	<27.8	56	35.7
Overweight	158	44.3	28	30.4	15	41.7	72	45.9
Obese	74	20.7	21	22.8	14	38.9	29	18.5
Chronic diseases								
Cardiovascular disease	51	14.3	11	12.0	<10	<27.8	17	10.8
Lung disease	52	14.6	13	14.1	11	30.6	12	7.6
Diabetes	15	4.2	<10	<10.9	<10	<27.8	<10	<6.4
Chronic muscle disease	<10	<2.8	<10	<10.9	<10	<27.8	<10	<6.4
Autoimmune disease	14	3.9	11	12.0	<10	<27.8	<10	<6.4
Psychiatric disorder	14	3.9	<10	<10.9	<10	<27.8	<10	<6.4
Other chronic disease	75	21.0	29	31.5	<10	<27.8	27	17.2
Chronic disease								
No chronic disease	214	59.9	47	51.1	16	44.4	112	71.3
One chronic disease	86	24.1	24	26.1	<10	<27.8	29	18.5
Multimorbidity	57	16.0	21	22.8	12	33.3	16	10.2
Vaccination status								
Fully vaccinated	166	46.5	35	38.0	11	30.6	42	26.8
Partially vaccinated	15	4.2	0	0.0	0	0.0	<10	<6.4
Not vaccinated	176	49.3	57	62.0	25	69.4	112	71.3
Likely virus variant								
Omicron	282	79.0	64	69.6	24	66.7	106	67.5
Delta	59	16.5	25	27.2	<10	<27.8	49	31.2
Alpha	16	4.5	<10	<10.9	<10	<27.8	<10	<6.4
Hospitalization								
No	331	92.7	87	94.6	30	83.3	155	98.7
Yes	26	7.3	<10	<10.9	6	16.7	<10	<6.4
Season of infection								
Winter	263	73.7	61	66.3	24	66.7	105	66.9
Spring	40	11.2	15	16.3	<10	<27.8	16	10.2
Summer	<10	<2.8	<10	<10.9	<10	<27.8	<10	<6.4
Autumn	46	12.9	15	16.3	<10	<27.8	34	21.7

Supplement table 7. Mean (SD) symptom severity, presence of symptoms at moderate severity, and presence of symptoms with a substantial increase of symptom severity (i.e. 1-point increase) to at least moderate severity 90-150 days after infection in those with COVID-19 and post COVID-19 condition

	**COVID-19 (n=2,441)**			**Post COVID-19 condition (n=1,024)**				
	**Mean (SD)**	**Presences of symptoms**		**Mean (SD)**	**Presences of symptoms**		**Substantial increase of symptom severity**	
		**n**	**%**		**n**	**%**	**n**	**%**
Headache	1.36 (0.52)	35	1.4	1.96 (0.99)	217	21.2	166	16.2
Dizziness	1.15 (0.35)	<10	<0.4	1.50 (0.75)	86	8.4	75	7.3
Chest pain	1.08 (0.26)	<10	<0.4	1.31 (0.61)	57	5.6	51	5.0
Lower back pain	1.37 (0.55)	63	2.6	1.98 (1.10)	272	26.6	191	18.7
Nausea	1.13 (0.34)	<10	<0.4	1.47 (0.76)	88	8.6	72	7.0
Painful muscles	1.41 (0.56)	71	2.9	2.15 (1.06)	322	31.4	242	23.6
Difficulties breathing	1.07 (0.26)	<10	<0.4	1.37 (0.70)	73	7.1	64	6.3
Feeling hot and cold alternately	1.15 (0.37)	13	0.5	1.66 (0.97)	167	16.3	134	13.1
Tingling extremities	1.15 (0.37)	20	0.8	1.52 (0.84)	124	12.1	100	9.8
Feeling a lump in the throat	1.09 (0.29)	<10	<0.4	1.34 (0.71)	64	6.3	59	5.8
General tiredness	1.15 (0.36)	<10	<0.4	1.78 (0.97)	184	18.0	164	16.0
Heavy arms and legs	1.13 (0.34)	<10	<0.4	1.70 (0.95)	174	17.0	143	14.0
Pain when breathing	1.02 (0.13)	0	0.0	1.14 (0.46)	22	2.1	22	2.1
Runny nose	1.33 (0.48)	22	0.9	1.77 (0.92)	183	17.9	133	13.0
Sore throat	1.13 (0.33)	<10	<0.4	1.46 (0.78)	98	9.6	88	8.6
Dry cough	1.19 (0.40)	<10	<0.4	1.52 (0.80)	102	10.0	91	8.9
Wet cough	1.15 (0.36)	10	0.4	1.41 (0.79)	101	9.9	86	8.4
Fever	1.01 (0.09)	0	0.0	1.10 (0.41)	20	2.0	18	1.8
Diarrhoea	1.09 (0.29)	<10	<0.4	1.28 (0.63)	50	4.9	43	4.2
Stomach pain	1.12 (0.32)	<10	<0.4	1.40 (0.70)	77	7.5	58	5.7
Ageusia/anosmia	1.07 (0.29)	<10	<0.4	1.62 (1.17)	191	18.7	185	18.1
Itchy eyes	1.17 (0.37)	11	0.5	1.59 (0.88)	142	13.9	106	10.4
Sneezing	1.42 (0.50)	20	0.8	1.80 (0.81)	165	16.1	111	10.8

Supplement table 8. Participant characteristics of the analytic study sample (n=3,465)

	**Total study Sample (n=3,465)**		**COVID-19 (n=2,441)**		**Post COVID-19 Condition (n=1,024)**	
**Characteristic**	n	%	n	%	n	%
Age						
18-39	343	9.9	218	8.9	125	12.2
40-59	1,810	52.2	1,234	50.6	576	56.3
≥60	1,312	37.9	989	40.5	323	31.5
Sex						
Male	1,238	35.7	969	39.7	269	26.3
Female	2,227	64.3	1,472	60.3	755	73.7
Educational level						
High	1,185	34.2	820	33.6	365	35.6
Medium	1,380	39.8	944	38.7	436	42.6
Low	811	23.4	614	25.2	197	19.2
Unknown	89	2.6	63	2.6	26	2.5
Smoking						
No	3,132	90.4	2,208	90.5	924	90.2
Yes	333	9.6	233	9.5	100	9.8
Body Mass index						
Healthy	1,535	44.3	1,129	46.3	406	39.6
Overweight	1,386	40.0	968	39.7	418	40.8
Obese	544	15.7	344	14.1	200	19.5
Chronic diseases						
Cardiovascular disease	345	10.0	221	9.1	124	12.1
Lung disease	336	9.7	209	8.6	127	12.4
Diabetes	88	2.5	51	2.1	37	3.6
Chronic muscle disease	46	1.3	20	0.8	26	2.5
Autoimmune disease	145	4.2	91	3.7	54	5.3
Psychiatric disorder	76	2.2	45	1.8	31	3.0
Other chronic disease	523	15.1	321	13.2	202	19.7
Chronic disease						
No chronic disease	2,469	71.3	1,811	74.2	658	64.3
One chronic disease	601	17.3	383	15.7	218	21.3
Multimorbidity	395	11.4	247	10.1	148	14.5
Vaccination status						
Fully vaccinated	1,584	45.7	1,107	45.4	477	46.6
Partially vaccinated	98	2.8	69	2.8	29	2.8
Not vaccinated	1,783	51.5	1,265	51.8	518	50.6
Virus variant						
Omicron	2,656	76.7	1,848	75.7	808	78.9
Delta	649	18.7	469	19.2	180	17.6
Alpha	160	4.6	124	5.1	36	3.5
Hospitalization						
No	3,340	96.4	2,360	96.7	980	95.7
Yes	125	3.6	81	3.3	44	4.3
Season of infection						
Winter	2,555	73.7	1,798	73.7	757	73.9
Spring	388	11.2	271	11.1	117	11.4
Summer	77	2.2	55	2.3	22	2.1
Autumn	445	12.8	317	13.0	128	12.5

Supplement table 9. Fit statistics for the Latent Profile Analysis

Model	Classes	AIC	BIC	Entropy	Minimal probability	Maximal probability	N min	N max	BLRT-p
1	1	55950.33	56177.18	1.00	1.00	1.00	1.00	1.00	
1	2	53822.97	54168.17	0.91	0.94	0.98	0.20	0.80	0.01
1	3	53355.66	53819.22	0.94	0.93	0.98	0.14	0.71	0.01
1	4	52479.45	53061.37	0.94	0.90	0.99	0.05	0.65	0.01
1	5	51917.13	52617.40	0.93	0.91	0.99	0.03	0.59	0.01
1	6	51408.16	52226.78	0.93	0.90	0.99	0.03	0.54	0.01
1	7	51410.30	52347.28	0.91	0.73	0.99	0.02	0.49	0.15
1	8	51215.59	52270.92	0.88	0.72	0.98	0.03	0.41	0.01
1	9	50832.60	52006.29	0.89	0.76	0.99	0.02	0.38	0.01
1	10	50625.29	51917.33	0.89	0.72	1.00	0.02	0.37	0.01
2	1	51444.89	52919.40	1.00	1.00	1.00	1.00	1.00	
2	2	50389.76	51982.63	1.00	1.00	1.00	0.07	0.93	0.01
2	3	49548.61	51259.83	0.99	1.00	1.00	0.06	0.76	0.01
2	4	49486.17	51315.74	0.90	0.82	1.00	0.06	0.61	0.01
2	5	49264.90	51212.83	0.91	0.87	1.00	0.05	0.54	0.01
2	6	49285.71	51352.00	0.85	0.70	1.00	0.06	0.47	0.41
2	7	49099.29	51283.93	0.87	0.78	1.00	0.06	0.42	0.01
2	8	49030.66	51333.65	0.84	0.74	1.00	0.04	0.33	0.01
2	9	48841.90	51263.25	0.87	0.75	1.00	0.04	0.36	0.01
2	10	48491.42	51031.13	0.87	0.72	1.00	0.03	0.35	0.01
3	Could not be estimated								
4	Could not be estimated								

Model 1: variance within class, no variance between classes, no covariance within class, no covariance between classes
Model 2: variance within class, no variance between classes, covariance within class, no covariance between classes
Model 3: variance within class, variance between classes, no covariance within class, no covariance between classes
Model 4: variance within class, variance between classes, covariance within class, covariance between classes
Abbreviations: AIC: Akaike information criterion; BIC: Bayesian information criterion; BLRT: bootstrapped likelihood test

Supplement table 10. Mean (SD) symptom severity and presence of symptoms with a substantial increase of symptom severity (i.e. 1-point increase) to at least moderate severity 90-150 days after infection by symptom profile

	No post COVID-19 (n=2,441)	Unspecific symptoms (n=662)			Fatigue (n=151)			Cardiorespiratory (n=52)			Ageusia/anosmia (n=159)		
	Mean (SD)	Mean (SD)	Substantial increase of symptom severity		Mean (SD)	Substantial increase of symptom severity		Mean (SD)	Substantial increase of symptom severity		Mean (SD)	Substantial increase of symptom severity	
			n	%		n	%		n	%		n	%
Headache	1.36 (0.52)	1.86 (0.89)	92	13.9	2.68 (1.18)	51	33.8	2.44 (1.04)	13	25.0	1.54 (0.75)	10	6.3
Dizziness	1.15 (0.35)	1.39 (0.66)	40	6.0	2.07 (0.96)	27	17.9	1.95 (0.79)	<10	<19.2	1.28 (0.50)	<10	<6.3
Chest pain	1.08 (0.26)	1.17 (0.42)	13	2.0	1.49 (0.62)	<10	<6.6	2.84 (0.89)	28	53.8	1.19 (0.40)	<10	<6.3
Lower back pain	1.37 (0.55)	1.97 (1.06)	141	21.3	2.12 (1.20)	20	13.2	2.46 (1.37)	15	28.8	1.73 (0.96)	15	9.4
Nausea	1.13 (0.34)	1.34 (0.62)	32	4.8	2.14 (1.05)	33	21.9	1.77 (0.78)	<10	<19.2	1.30 (0.59)	<10	<6.3
Painful muscles	1.41 (0.56)	2.00 (1.00)	148	22.4	2.73 (1.12)	48	31.8	3.13 (0.87)	24	46.2	1.88 (0.87)	22	13.8
Difficulties breathing	1.07 (0.26)	1.22 (0.51)	20	3.0	1.51 (0.62)	<10	<6.6	3.19 (0.87)	34	65.4	1.27 (0.50)	<10	<6.3
Feeling hot/cold alternately	1.15 (0.37)	1.48 (0.80)	64	9.7	2.49 (1.18)	47	31.1	2.34 (1.27)	17	32.7	1.38 (0.71)	<10	<6.3
Tingling extremities	1.15 (0.37)	1.42 (0.75)	56	8.5	1.89 (1.00)	22	14.6	2.08 (1.10)	13	25.0	1.41 (0.79)	<10	<6.3
Feeling a lump in the throat	1.09 (0.29)	1.21 (0.52)	19	2.9	1.85 (1.05)	29	19.2	1.72 (1.05)	<10	<19.2	1.26 (0.60)	<10	<6.3
General tiredness	1.15 (0.36)	1.50 (0.67)	47	7.1	2.98 (0.99)	82	54.3	2.94 (1.22)	23	44.2	1.45 (0.73)	12	7.5
Heavy arms and legs	1.13 (0.34)	1.43 (0.68)	46	6.9	2.77 (1.11)	68	45.0	2.70 (1.18)	18	34.6	1.45 (0.71)	11	6.9
Pain when breathing	1.02 (0.13)	1.05 (0.22)	<10	<1.5	1.16 (0.35)	0	0.0	2.58 (0.90)	21	40.4	1.05 (0.21)	0	0.0
Runny nose	1.33 (0.48)	1.70 (0.84)	77	11.6	2.20 (1.13)	36	23.8	1.91 (0.99)	<10	<19.2	1.64 (0.85)	11	6.9
Sore throat	1.13 (0.33)	1.36 (0.68)	44	6.6	1.91 (0.97)	27	17.9	1.88 (0.18)	10	19.2	1.29 (0.57)	<10	<6.3
Dry cough	1.19 (0.40)	1.48 (0.78)	56	8.5	1.83 (0.95)	25	16.6	1.84 (1.04)	<10	<19.2	1.26 (0.52)	<10	<6.3
Wet cough	1.15 (0.36)	1.35 (0.70)	48	7.3	1.71 (0.99)	25	16.6	1.86 (1.14)	<10	<19.2	1.24 (0.64)	<10	<6.3
Fever	1.01 (0.09)	1.05 (0.28)	<10	<1.5	1.33 (0.69)	<10	<6.6	1.25 (0.68)	<10	<19.2	1.04 (0.24)	<10	<6.3
Diarrhoea	1.09 (0.29)	1.19 (0.50)	20	3.0	1.70 (0.92)	15	9.9	1.59 (0.97)	<10	<19.2	1.16 (0.38)	<10	<6.3
Stomach pain	1.12 (0.32)	1.28 (0.57)	26	3.9	1.97 (1.00)	24	15.9	1.81 (0.92)	<10	<19.2	1.20 (0.42)	<10	<6.3
Ageusia/anosmia	1.07 (0.29)	1.07 (0.25)	0	0.0	1.54 (0.93)	20	13.2	1.94 (1.39)	12	23.1	3.89 (0.83)	153	96.2
Itchy eyes	1.17 (0.37)	1.51 (0.82)	68	10.3	2.01 (1.09)	27	17.9	2.05 (1.00)	<10	<19.2	1.36 (0.68)	<10	<6.3
Sneezing	1.42 (0.50)	1.75 (0.78)	69	10.4	2.18 (0.98)	28	18.5	2.01 (0.80)	<10	<19.2	1.57 (0.66)	<10	<6.3

Supplement table 11. Participant characteristics per symptom profile

	**Unspecific symptoms (n=662)**		**Fatigue (n=151)**		**Cardiorespiratory (n=52)**		**Ageusia / anosmia (n=159)**	
	N	%	N	%	N	%	n	%
Age								
18-39	89	13.4	17	11.3	<10	<19.2	15	9.4
40-59	359	54.2	93	61.6	32	61.5	92	57.9
≥60	214	32.3	41	27.2	16	30.8	52	32.7
Sex								
Male	171	25.8	29	19.2	14	26.9	55	34.6
Female	491	74.2	122	80.8	38	73.1	104	65.4
Educational level								
High	238	36.0	42	27.8	22	42.3	63	39.6
Medium	279	42.1	74	49.0	21	40.4	62	39.0
Low	127	19.2	34	22.5	<10	<19.2	28	17.6
Unknown	18	2.7	<10	<6.6	<10	<19.2	<10	<6.3
Smoking								
No	601	90.8	136	90.1	47	90.4	140	88.1
Yes	61	9.2	15	9.9	<10	<19.2	19	11.9
Body Mass index								
Healthy	274	41.4	58	38.4	14	26.9	60	37.7
Overweight	270	40.8	57	37.7	21	40.4	70	44.0
Obese	118	17.8	36	23.8	17	32.7	29	18.2
Chronic diseases								
Cardiovascular disease	82	12.4	14	9.3	11	21.2	17	10.7
Lung disease	74	11.2	25	16.6	16	30.8	12	7.5
Diabetes	22	3.3	<10	<6.6	<10	<19.2	<10	<6.3
Chronic muscle disease	<10	<1.5	<10	<6.6	<10	<19.2	<10	<6.3
Autoimmune disease	31	4.7	11	7.3	10	19.2	<10	<6.3
Psychiatric disorder	12	1.8	10	6.6	<10	<19.2	<10	<6.3
Other chronic disease	118	17.8	45	29.8	11	21.2	28	17.6
Chronic diseases								
No chronic disease	437	66.0	85	56.3	24	46.2	112	70.4
One chronic disease	139	21.0	37	24.5	12	23.1	30	18.9
Multimorbidity	86	13.0	29	19.2	16	30.8	17	10.7
Vaccination status								
Fully vaccinated	352	53.2	70	46.4	16	30.8	39	24.5
Partially vaccinated	21	3.2	<10	<6.6	<10	<19.2	<10	<6.3
Not vaccinated	289	43.7	77	51.0	35	67.3	117	73.6
Likely virus variant								
Omicron	554	83.7	112	74.2	34	65.4	108	67.9
Delta	85	12.8	34	22.5	12	23.1	49	30.8
Alpha	23	3.5	<10	<6.6	<10	<19.2	<10	<6.3
Hospitalization								
No	640	96.7	141	93.4	45	86.5	154	96.9
Yes	22	3.3	10	6.6	<10	<19.2	<10	<6.3
Season of infection								
Winter	514	77.6	104	68.9	34	65.4	105	66.0
Spring	74	11.2	16	10.6	<10	<19.2	18	11.3
Summer	13	2.0	<10	<6.6	<10	<19.2	<10	<6.3
Autumn	61	9.2	26	17.2	<10	<19.2	34	21.4

Supplement table 12. Univariable and multivariable associations between participant characteristics and post COVID-19 condition

		Univariable	Multivariable
	n	OR (95% CI)	OR (95% CI)
Age			
18-39	343	Ref	Ref
40-59	1,810	0.81 (0.64, 1.04)	0.75 (0.58, 0.97)
≥60	1,312	0.57 (0.44, 0.73)	0.55 (0.42, 0.72)
Sex			
Male	1,238	Ref	Ref
Female	2,227	1.85 (1.57, 2.17)	1.79 (1.51, 2.12)
Educational level			
High	1,185	Ref	Ref
Medium	1,380	1.04 (0.88, 1.23)	0.93 (0.78, 1.11)
Low	811	0.72 (0.59, 0.88)	0.68 (0.55, 0.85)
Unknown	89	0.93 (0.58, 1.49)	0.74 (0.45, 1.24)
Smoking			
No	3,132	Ref	Ref
Yes	333	1.03 (0.80, 1.31)	0.96 (0.74, 1.24)
Body Mass index			
Healthy	1,535	Ref	Ref
Overweight	1,386	1.20 (1.02, 1.41)	1.31 (1.10, 1.54)
Obese	544	1.62 (1.31, 1.99)	1.49 (1.19, 1.86)
Cardiovascular disease			
No	3,120	Ref	Ref
Yes	345	1.38 (1.10, 1.75)	1.27 (0.98, 1.65)
Lung disease			
No	3,129	Ref	Ref
Yes	336	1.51 (1.20, 1.91)	1.22 (0.95, 1.57)
Diabetes			
No	3,377	Ref	Ref
Yes	88	1.76 (1.14, 2.70)	1.63 (1.03, 2.58)
Chronic muscle disease			
No	3,419	Ref	Ref
Yes	46	3.15 (1.75, 5.67)	2.20 (1.18, 4.10)
Autoimmune disease			
No	3,320	Ref	Ref
Yes	145	1.44 (1.02, 2.03)	1.05 (0.72, 1.52)
Psychiatric disorder			
No	3,389	Ref	Ref
Yes	76	1.66 (1.05, 2.64)	1.13 (0.69, 1.84)
Other chronic disease			
No	2,942	Ref	Ref
Yes	523	1.62 (1.34, 1.97)	1.41 (1.14, 1.74)
Vaccination status			
Fully vaccinated	1,584	Ref	Ref
Partially vaccinated	98	0.97 (0.62, 1.52)	0.89 (0.56, 1.42)
Not vaccinated	1,783	0.95 (0.82, 1.10)	0.96 (0.80, 1.17)
Virus variant			
Omicron	2,656	Ref	Ref
Delta	649	0.88 (0.72, 1.06)	0.84 (0.63, 1.13)
Alpha	160	0.66 (0.45, 0.97)	0.41 (0.25, 0.67)
Hospitalization			
No	3,340	Ref	Ref
Yes	125	1.31 (0.90, 1.90)	1.67 (1.09, 2.54)
Season of infection			
Winter	2,555	Ref	Ref
Spring	388	1.02 (0.81, 1.29)	1.23 (0.90, 1.67)
Summer	77	0.95 (0.57, 1.57)	0.89 (0.52, 1.53)
Autumn	445	0.96 (0.77, 1.20)	1.05 (0.77, 1.41)

Supplement table 13. Multivariable multinomial logistic regression analysis of determinants for profile status among participants with COVID-19 condition*

	Unspecific symptoms (n=644)	Fatigue (n=150)	Cardiorespiratory (n=51)	Ageusia/anosmia (n=153)
	OR (95% CI)	OR (95% CI)	OR (95% CI)	OR (95% CI)
Age				
18-39	Ref	Ref	Ref	Ref
40-59	0.70 (0.52, 0.94)	0.85 (0.48, 1.51)	1.37 (0.40, 4.69)	1.05 (0.57, 1.95)
≥60	0.53 (0.39, 0.74)	0.48 (0.25, 0.91)	0.89 (0.24, 3.31)	0.92 (0.47, 1.77)
Sex				
Male	Ref	Ref	Ref	Ref
Female	1.83 (1.49, 2.23)	2.39 (1.56, 3.65)	1.47 (0.77, 2.82)	1.30 (0.91, 1.85)
Educational level				
High	Ref	Ref	Ref	Ref
Medium	0.94 (0.77, 1.16)	1.33 (0.89, 1.99)	0.59 (0.31, 1.12)	0.73 (0.50, 1.06)
Low	0.70 (0.54, 0.90)	1.02 (0.62, 1.67)	0.31 (0.13, 0.76)	0.50 (0.31, 0.81)
Smoking				
No	Ref	Ref	Ref	Ref
Yes	0.86 (0.63, 1.18)	1.00 (0.57, 1.76)	0.84 (0.31, 2.31)	1.34 (0.80, 2.27)
Body Mass index				
Healthy	Ref	Ref	Ref	Ref
Overweight	1.23 (1.01, 1.50)	1.21 (0.82, 1.78)	1.75 (0.85, 3.59)	1.50 (1.04, 2.18)
Obese	1.39 (1.07, 1.80)	1.61 (1.01, 2.57)	2.92 (1.29, 6.60)	1.72 (1.05, 2.81)
Cardiovascular disease				
No	Ref	Ref	Ref	Ref
Yes	1.46 (1.09, 1.97)	0.69 (0.37, 1.29)	1.36 (0.60, 3.09)	1.03 (0.58, 1.85)
Lung disease				
No	Ref	Ref	Ref	Ref
Yes	1.15 (0.85, 1.56)	1.44 (0.88, 2.36)	3.11 (1.55, 6.27)	0.82 (0.43, 1.54)
Diabetes				
No	Ref	Ref	Ref	Ref
Yes	1.50 (0.88, 2.57)	2.52 (1.09, 5.83)	1.17 (0.25, 5.58)	1.46 (0.55, 3.90)
Chronic muscle disease				
No	Ref	Ref	Ref	Ref
Yes	1.25 (0.55, 2.85)	3.50 (1.40, 8.76)	5.40 (1.77, 16.5)	1.73 (0.38, 7.86)
Autoimmune disease				
No	Ref	Ref	Ref	Ref
Yes	1.12 (0.72, 1.74)	1.26 (0.62, 2.55)	3.93 (1.65, 9.34)	0.29 (0.07, 1.22)
Psychiatric disorder				
No	Ref	Ref	Ref	Ref
Yes	0.69 (0.35, 1.37)	2.02 (0.94, 4.35)	2.82 (0.90, 8.84)	1.08 (0.37, 3.14)
Other chronic disease				
No	Ref	Ref	Ref	Ref
Yes	1.31 (1.02, 1.68)	2.20 (1.47, 3.31)	0.85 (0.40, 1.83)	1.60 (1.01, 2.54)
Vaccination status				
Fully vaccinated	Ref	Ref	Ref	Ref
Not vaccinated	0.81 (0.65, 1.01)	0.76 (0.49, 1.19)	1.50 (0.71, 3.20)	2.70 (1.76, 4.14)
Virus variant				
Omicron	Ref	Ref	Ref	Ref
Delta	0.73 (0.51, 1.06)	1.11 (0.57, 2.14)	0.93 (0.33, 2.66)	0.94 (0.54, 1.64)
Alpha	0.44 (0.25, 0.79)	0.36 (0.11, 1.13)	1.66 (0.38, 7.19)	0.13 (0.03, 0.60)
Hospitalization				
No	Ref	Ref	Ref	Ref
Yes	1.47 (0.86, 2.50)	2.74 (1.26, 5.97)	3.00 (1.08, 8.29)	1.30 (0.49, 3.46)
Season of infection				
Winter	Ref	Ref	Ref	Ref
Spring	1.31 (0.91, 1.88)	1.24 (0.61, 2.51)	0.73 (0.21, 2.53)	1.11 (0.60, 2.05)
Summer	0.91 (0.48, 1.75)	0.88 (0.28, 2.77)	1.01 (0.20, 5.20)	0.45 (0.10, 1.98)
Autumn	0.88 (0.60, 1.29)	1.40 (0.75, 2.61)	1.05 (0.35, 3.17)	1.30 (0.73, 2.29)

* Due to a low number of participants in some cells: unknown education excluded; partly vaccinated combined with fully vaccinated

Supplement table 14. Multivariable multinomial logistic regression analysis of determinants for profile status with the muscle pain profile (n=349) as the reference group*

	**Fatigue (n=92)**	**Cardiorespiratory (n=35)**	**Ageusia/anosmia (n=151)**
	OR (95% CI)	OR (95% CI)	OR (95% CI)
Age			
18-39	Ref	Ref	Ref
40-59	0.92 (0.40, 1.13)	3.62 (0.46, 28.4)	1.43 (0.66, 3.09)
≥60	0.99 (0.40, 2.42)	1.78 (0.20, 15.6)	1.43 (0.63, 3.25)
Sex			
Male	Ref	Ref	Ref
Female	1.43 (0.80, 2.56)	1.09 (0.47, 2.49)	0.73 (0.48, 1.13)
Educational level			
High	Ref	Ref	Ref
Medium	0.98 (0.57, 1.71)	0.40 (0.18, 0.90)	0.73 (0.46, 1.14)
Low	1.08 (0.56, 2.10)	0.31 (0.10, 0.92)	0.60 (0.34, 1.08)
Smoking			
No	Ref	Ref	Ref
Yes	2.32 (1.10, 4.89)	1.80 (0.55, 5.93)	1.41 (0.70, 2.83)
Body Mass index			
Healthy	Ref	Ref	Ref
Overweight	0.49 (0.28, 0.86)	2.21 (0.80, 6.13)	1.10 (0.69, 1.74)
Obese	0.72 (0.38, 1.36)	3.62 (1.23, 10.6)	1.10 (0.62, 1.98)
Chronic disease			
No	Ref	Ref	Ref
One	1.36 (0.76, 2.42)	1.13 (0.44, 2.86)	0.66 (0.39, 1.09)
Multimorbidity	1.76 (0.93, 3.33)	2.51 (1.02, 6.18)	0.57 (0.30, 1.08)
Vaccination status			
Fully vaccinated	Ref	Ref	Ref
Not vaccinated	1.27 (0.69, 2.32)	2.27 (0.91, 5.68)	2.44 (1.49, 4.00)
Virus variant			
Omicron	Ref	Ref	Ref
Delta	2.17 (0.97, 4.85)	0.81 (0.23, 2.92)	1.22 (0.63, 2.36)
Alpha	0.65 (0.15, 2.76)	2.54 (0.38, 17.0)	0.26 (0.05, 1.33)
Season			
Winter	Ref	Ref	Ref
Spring/summer	1.37 (0.63, 2.97)	0.50 (0.10, 2.43)	0.78 (0.38, 1.61)
Autumn	0.80 (0.34, 1.86)	1.33 (0.36, 4.87)	1.14 (0.57, 2.26)

* Due to a low number of participants in some cells: unknown education excluded; partly vaccinated combined with fully vaccinated; spring and summer combined
